# Modeling human early otic sensory cell development with induced pluripotent stem cells

**DOI:** 10.1371/journal.pone.0198954

**Published:** 2018-06-14

**Authors:** Hanae Lahlou, Alejandra Lopez-Juarez, Arnaud Fontbonne, Emmanuel Nivet, Azel Zine

**Affiliations:** 1 Aix Marseille Université, CNRS, LNIA UMR 7260, Marseille, France; 2 Aix Marseille Université, CNRS, NICN UMR 7259, Marseille, France; 3 Université de Montpellier, Faculté de Pharmacie, Montpellier, France; Texas A&M University, UNITED STATES

## Abstract

The inner ear represents a promising system to develop cell-based therapies from human induced pluripotent stem cells (hiPSCs). In the developing ear, Notch signaling plays multiple roles in otic region specification and for cell fate determination. Optimizing hiPSC induction for the generation of appropriate numbers of otic progenitors and derivatives, such as hair cells, may provide an unlimited supply of cells for research and cell-based therapy. In this study, we used monolayer cultures, otic-inducing agents, Notch modulation, and marker expression to track early and otic sensory lineages during hiPSC differentiation. Otic/placodal progenitors were derived from hiPSC cultures in medium supplemented with FGF3/FGF10 for 13 days. These progenitor cells were then treated for 7 days with retinoic acid (RA) and epidermal growth factor (EGF) or a Notch inhibitor. The differentiated cultures were analyzed in parallel by qPCR and immunocytochemistry. After the 13 day induction, hiPSC-derived cells displayed an upregulated expression of a panel of otic/placodal markers. Strikingly, a subset of these induced progenitor cells displayed key-otic sensory markers, the percentage of which was increased in cultures under Notch inhibition as compared to RA/EGF-treated cultures. Our results show that modulating Notch pathway during *in vitro* differentiation of hiPSC-derived otic/placodal progenitors is a valuable strategy to promote the expression of human otic sensory lineage genes.

## Introduction

Hearing loss and vestibular dysfunction are the most common sensory deficits in humans [1]. The inner ear is a highly specialized sensory organ containing auditory and vestibular hair cells (HCs) that transduce mechanical energy into electrical energy for transmission to the central nervous system [2]. During otic development, HCs in the inner ear are derived from the differentiation of early otic progenitor cells through a precise temporally and spatially-coordinated pattern of gene expression orchestrated by complex signaling cascades [3^_^^4^]. A normal human cochlea contains approximately 16,000 sensory HCs forming one row of inner HCs and three rows of outer HCs. They are limited in number and are susceptible to damage from a variety of insults, ranging from ototoxic drugs to loud noise exposure, genetic mutations, or the effects of aging. In contrast to the avian cochlea able to regenerate lost HCs [5–6], the mature mammalian cochlea is unable to spontaneously regenerate HCs leading to permanent hearing loss.

Over the past few years, stem cell-based therapy approaches aiming to emulate otic development in the production of HCs from stem cells have received substantial interest [7–8]. The generation of replacement HCs from a renewable source of otic progenitors remains one of the principal requirements for the successful development of a cell-based therapy within the inner ear. Murine embryonic stem cells (mESCs) have already demonstrated their capability of differentiating into otic epithelial lineage *in vitro* [9–15]. Furthermore, previous studies with human embryonic stem cells (hESCs) have revealed their ability to differentiate along an otic neurogenic lineage, giving rise to neurons with a partial functional restoration of HC innervation in an animal model of auditory neuropathy [16–17].

There is also evidence that hESCs are able to differentiate into cells of otic epithelial lineage when grown in aggregate/embryoid body (EB)- or adherent cell cultures [18–19]. Recently, the concept of differentiating hESC-derived HC-like cells has been elegantly demonstrated by the ability of these hESCs to differentiate self-guided when cultured in hydrogels as extracellular matrix mimics for three-dimensional (3D) cell culture [20]. These EB/aggregate and 3D-organoid *in vitro* guidance methods did allow the generation of HC-like cells displaying stereocilia bundles from pluripotent stem cells. However, they were found to be complex and time-consuming with variable efficiency and were not appropriate for the isolation of dissociated otic progenitors required for the development of cell-based therapies.

Human ESCs challenged with retinoic acid (RA), epidermal growth factor (EGF), and other growth factors have previously been shown to differentiate into HC-like cells [17]. However, this study was mainly focused on otic neural progenitors and thus did not explain or characterize the presumptive otic/placodal progenitors. The available differentiation protocols remain unsatisfactory and require further investigation in order to obtain higher yields of otic sensory progenitors. Despite enormous progress made towards unraveling the signaling cascades governing otic sensory differentiation, and their sequential orchestration during development, much of otic cell fate determination remains not fully understood yet. The key to the *in vitro* production of otic/placodal progenitors and their further differentiation into human otic sensory cells is the identification of critical developmental pathways, as well as how and when they have to be modulated.

In this line, human pluripotent stem cells, either from embryonic origin [21] or obtained by cell reprogramming [22], namely human induced pluripotent stem cells (hiPSCs), have received considerable attention for use as a cellular platform for the *in vitr*o production of cell types of interest, including otic cells, by recapitulating their developmental steps. Indeed, thanks to their self-renewal and pluripotency features, they represent a suitable source for *in vitro* generation of a large number of otic progenitors and their derivatives, including initial HCs. Moreover, in the future, hiPSCs may serve as an autologous source of replacement for HCs and/or neurons in the injured inner ear, if their differentiation to early otic/placodal progenitors and otic sensory lineage is first successful *in vitro*.

Among the molecular pathways that regulate otic specification and competence during development, the Notch pathway is a critical player [23–28]. Numerous studies have reported that Notch inhibition, in damaged cochleae, increases the conversion of adult stem cells to HCs by a mechanism involving the upregulation of *Atoh1* [29–30]. The Notch pathway is a highly conserved signaling pathway that regulates cell fate and development in a variety of metazoan tissues [31] and plays a crucial role in the regulation of stem cell behavior [32–33].

In this study, we investigated whether a disruption of the Notch pathway could affect the differentiation of hiPSC-derived otic/placodal cells into otic sensory cells as compared to RA/EGF treatment in monolayer cultures [17]. To monitor the generation of human otic sensory cell-lineage, we measured the expression of a comprehensive panel of early otic/placodal and sensory otic lineage markers in parallel using quantitative real time-PCR and immunocytochemistry analyses.

Our results show that timely inhibition of the Notch pathway during the differentiation of hiPSC-derived otic progenitor cells is a valuable strategy to efficiently generate human inner ear otic sensory cells *in vitro*. Our work improves our understanding of the mechanisms controlling otic sensory lineage differentiation and may contribute towards developing a cell-based therapy approach for inner ear disorders.

## Materials and methods

### Human induced pluripotent culture and maintenance method

The hiPSCs ChiPSC-4 line was provided by Cellartis (Göteborg, Sweden). This cell line was derived from fibroblasts of healthy human donors and reprogrammed by using polycistronic retrovirus technology, based on the transduction of Oct3/4, Sox2, Klf4, and c-Myc transcription factors [22, 34]. The hiPSCs were maintained using a proprietary feeder-free culture system, they were plated at a density of 40,000–50,000 cells/cm^2^ onto coated dishes with a DEF-CS^™^ COAT-1 matrix (1:20, Cellartis) diluted in D-PBS (+/+) (Gibco by Life Technologies). Cells were expanded in DEF-CS^™^ 500 basal medium, daily supplemented with DEF-CSTM GF-1 (1:333), GF-2 (1:1000) and GF-3 (1:1000) additives (Cellartis). When the cells were confluent at 80–90% (about 5–7 days), they were passaged using TrypLE Select^®^ (Life Technologies). The cells grew quickly, and in less than a week, we could proceed to the sof cells. Then, they were frozen or differentiated into inner ear otic cell lineage.

### Early otic placode induction

We performed initial otic placodal induction from hiPSCs using a monolayer culture system previously described for the differentiation of human embryonic stem cells (hESCs) into neuronal and epithelial cells [17]. In our study, undifferentiated hiPSCs, from passages 18–21, were seeded at 30,000 cells/cm^2^ onto coated laminin flasks (1.5 μg/cm^2^, R&D Systems). Cells were cultured in DFNB basal medium (DMEM/F12 with N2 and B27, Gibco by Life Technologies), supplemented with fibroblast growth factors, FGF3 (50 ng/ml) and FGF10 (50 ng/ml) (R&D Systems) from the first day until the end of the induction period (i.e. day 13). The medium was replaced every 2 days.

### Sensory otic cell differentiation

To induce differentiation into otic sensory cells, dissociated otic/placodal progenitors obtained from the early otic induction were transferred onto gelatin coated flasks at 80,000 cells/cm^2^ and cultured in DFNB medium, supplemented with either RA (1 mM, Sigma) and EGF (20 ng/ml; R&D Systems) or with a gamma-secretase inhibitor at 5 μM, i.e. difluorobenzeneacetamid (DBZ, Tocris Bioscience). Exposure to either RA/EGF or DBZ was initiated from day 14 to day 20 and the medium was replaced every 2 days. At different culture periods, qPCR and immunostaining were performed on differentiated cells and analyzed for the expression of early otic/placodal markers (i.e. *PAX2*, *PAX8*, *DLX5*, *GATA3*) and otic sensory markers (i.e. *ATOH1*, *POU4F3*, *MYO7A*).

### Quantitative RT-PCR

Total RNA was extracted from undifferentiated hiPSCs at day 0 and differentiated cells at day 13 and day 20 *in vitro* using the PureLink^®^ RNA Mini Kit (Life Technologies) according to manufacturer’s instructions. cDNA was synthesized from 1 μg of RNA per sample, using High-Capacity RNA-to-cDNA^™^ Kit (Life Technologies), and 5 μl of cDNA were submitted to qPCR reaction. Quantitative RT-PCR was performed with TaqMan^®^ Fast Real Time PCR System (Applied Biosystems). Primer pairs used are listed in S1 Table (in the supporting information). Samples were run in duplicates and analyzed with the 7500 Software v2.0 (Applied Biosystems). Relative expression levels were determined according to the ΔΔCt method, the *GAPDH* gene serving as endogenous control for normalization. RNA extractions were performed from three independent cultures and the reported values are the mean of these three independent experiments, each performed in duplicate.

All values are presented as the mean ± sem. Statistical differences between groups were analyzed by one-way ANOVA and the Student’s t-test using GraphPad Prism 6. Statistical difference was reported for p-values below 0.05, an asterisk indicates significant differences between means (*p < 0.05; ** p < 0.01; ***p < 0.001).

### Fluidigm assay

RNA was isolated from hiPSCs and differentiated cells as for standard qRT-PCR using the PureLink^®^ RNA Mini Kit (Life Technologies) according to manufacturer’s instructions [35]. We used 1 μg of total RNA from undifferentiated and differentiated cultures for cDNA synthesis using High-Capacity RNA-to-cDNA^™^ Kit (Life Technologies). For each sample, 6.5 ng cDNA was used to perform 14 cycles of pre-amplification using of TaqMan PreAmp Master Mix (Applied Biosystems) and a 96 pooled PCR primer mix (final 0.2μM of each). Pre-amplified PCR products were then treated with 4U of Exo I (NEB) in order to remove free primers resulting from pre-amplification. Finally, cleaned pre-amplification was diluted 1:5 with 1X TE Buffer. The qPCR experiments were performed using microfluidic 96.96 Dynamic Array chip and the BioMark^™^ HD System following manufacturer procedure. Final reaction volume of qPCR was 6.75 nL, with 500 nM primer final concentration and 1:18 sample final concentration. Primer pairs used are listed in S1 Table (in the supporting information). The data was acquired using Real-Time PCR Analysis Software in the BioMark instrument. Ct values were processed by automatic threshold for all assays, with derivative baseline correction using BioMark Real-Time PCR Analysis Software 4.1.3 (Fluidigm).

All data analyses were performed with R software v3.0 and ΔΔCt package v1.12. Data have been analyzed using the ΔΔCt package algorithm which was built on the 2^−(ΔΔCt)^ Method [36]. All values are presented as the mean ± sem. Statistical differences between groups were analyzed by one-way ANOVA and the Student’s t-test. Statistical difference was reported for p-values below 0.05, an asterisk indicates significant differences between means (*p < 0.05; ** p < 0.01; ***p < 0.001).

### Cochlea whole-mount surface preparation

The use of animals in this study was in accordance with the guidelines of CNRS (Centre National de la Recherche Scientifique). Animal housing and experiments were conducted in accordance with French national legislation (JO 87–848) and approved by our local ethics committee named “Direction Départementale de la Protection des Populations, Préfecture des Bouches du Rhône”, France (Permit No. B13-055-25).

The cochlear epithelium preparations were collected from postnatal day 1 Swiss wild-type mice. After decapitation, the temporal bones containing the inner ears were removed from the skull, the cochleae were opened and the sensory epithelium was separated from the modiolus by microdissection.

### Immunocytochemistry and microscopy

*In vitro* differentiated cells and cochlear epithelium whole mount-preparations were fixed with 4% paraformaldehyde in phosphate-buffered saline (PBS) for 15 min at room temperature. Unspecific binding was blocked in 0.3% Triton X-100, 10% normal donkey serum and 1% bovine serum albumin in PBS for 30 min. Then, samples were incubated overnight at 4°C with specific primary antibodies (S2 Table, in the supporting information) diluted in the same buffer without Triton X-100. They were then washed and incubated with AlexaFluor (Molecular Probes) secondary antibodies and the nuclei were counterstained with Hoechst. Control experiments including negative control (i.e. without primary antibodies) were processed in parallel. Samples were mounted using Prolong Gold Antifading reagent on Superfrost glass slides (Life Technologies). The images were acquired with a Zeiss confocal microscope LSM 710 NLO Zeiss and Zen software (Zeiss, Jena, Germany).

### Cell counting and statistical analysis

The cells were counted manually using Graphic tools of Zeiss computer software (Zen 2012, blue edition). The fraction of immuno+ cells among the total number of cells identified by Hoechst was used to label all nuclei in at least five fields per coverslip in each *in vitro* condition. Three independent experiments were conducted for each determination and data were expressed as mean ± sem. Data were analyzed using the Student’s t-test or one-way ANOVA. Statistical difference was reported for p-values below 0.05, an asterisk indicates significant differences between means (*p < 0.05; ** p < 0.01; ***p < 0.001).

## Results

### Efficient differentiation of hiPSC-derived otic/placodal progenitors

Placodal development and otic induction is a complex process involving multiple signaling steps during which, the specification and competence of individual cells become restricted until the progenitors are able to continue their differentiation towards lineage of defined cell types [37]. During otic development, many markers including non-neural ectoderm, preplacodal ectoderm, and early otic lineage genes are expressed in a fluctuating manner. Indeed some early gene markers are transiently down-regulated and later re-expressed, whereas, other genes are only transiently expressed or are continuously up-regulated during early otic development [38]. This pattern of gene expression may interfere with a clear chronological identification of cell populations during otic/placodal development in an *in vitro* environment. We therefore used the term “otic/placodal progenitors” to refer to all generated early otic lineage cell phenotypes in our *in vitro* differentiation.

**Fig 1:**
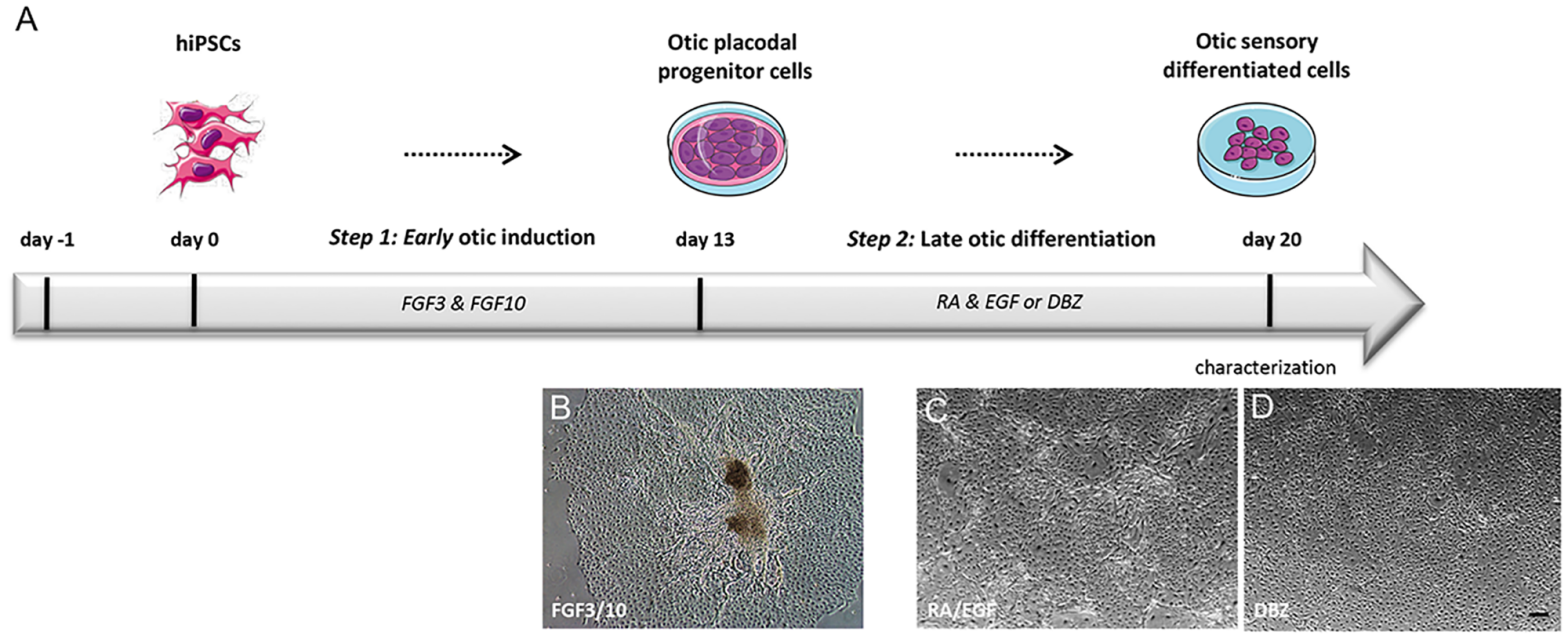
Schematic summary outlining the otic differentiation from hiPSCs in monolayer culture. (A) As a first step (step1), hiPSCs at day 0 were exposed to FGF3 and FGF10 growth factors until day 13 for early otic/placodal induction and, in a second step (step2) were then differentiated into otic sensory cells by exposure to either RA/EGF or DBZ until day 20. (B-D) Morphological characteristics of hiPSC-derived otic progenitor cells after FGF3/10, RA/EGF and DBZ treatments respectively. Scale bars, 200 μm. Abbreviations: DFNB, DMEM/F12 supplemented with N2 and B27; RA, retinoic acid; DBZ, difluoro-benzeneacetamide; EGF, epithelial growth factor; FGF, fibroblast growth factor.

**Fig 2:**
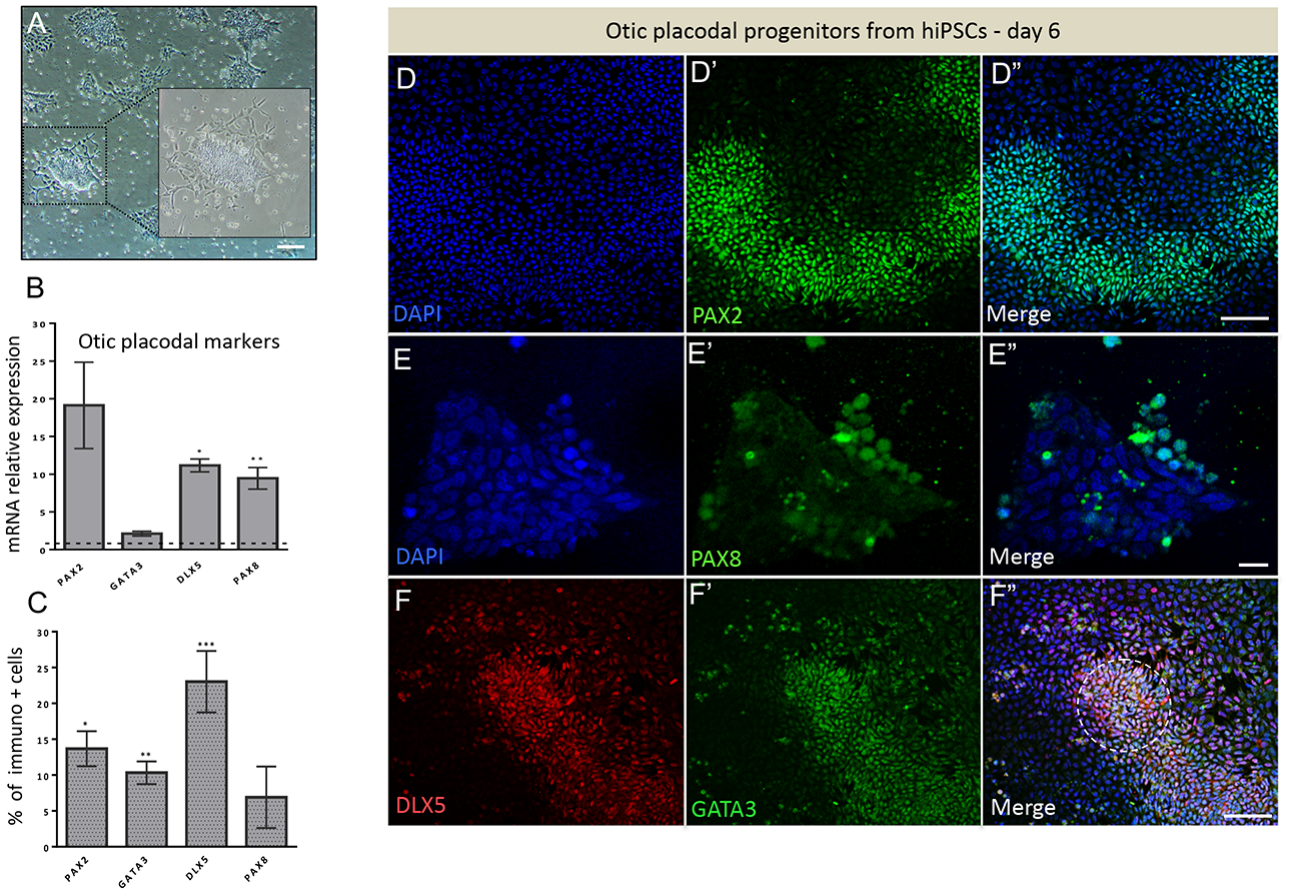
Development of otic/placodal competence and expression of early lineage markers in cultures at day 6 *in vitro*. (A) A bright-field view of hiPSC-derived otic/placodal progenitors after FGF3/10 exposure. An example of a colony displaying cells with spindle-like shape, indicating conversion efficiency from undifferentiated hiPSCs into early otic/placodal progenitors (insets). Scale bar, 200 μm. (B) A histogram depicting values that were expressed as fold change relative to hiPSCs using RT-qPCR. Data were normalized to control for the amount of RNA isolated from undifferentiated hiPSCs (day 0) and this level of mRNA expression was referred to as 1. At this stage of FGF treatment, a highly efficient induction of otic/placodal (i.e. *PAX2*, *PAX8* and *DLX5*) gene markers was observed as compared to undifferentiated hiPSCs. Statistical differences were determined with unpaired Student’s t-test when comparing mRNA expression levels between undifferentiated (day 0) and differentiated cells (day 6) cells. P values are indicated by *p < 0.05, **p < 0.01, and ***p < 0.001 (n = 3). (C) Quantification of the expression of early otic lineage markers by immunocytochemistry revealed their significant upregulation (PAX2, GATA3 and DLX5) in FGF-treated cultures as compared to in undifferentiated hiPSCs. (D-F) Representative immunostainings for PAX2, GATA3, PAX8 and DLX5 in day 6 cultures. A population of PAX2+, PAX8+ and GATA3+, DLX5+ cells are observed in these differentiated cultures. Interestingly, in some areas, cells co-expressed DLX5 and GATA3 (dotted circle, F). Hoechst staining is shown in blue. Scale bars, 50 μm.

**Fig 3:**
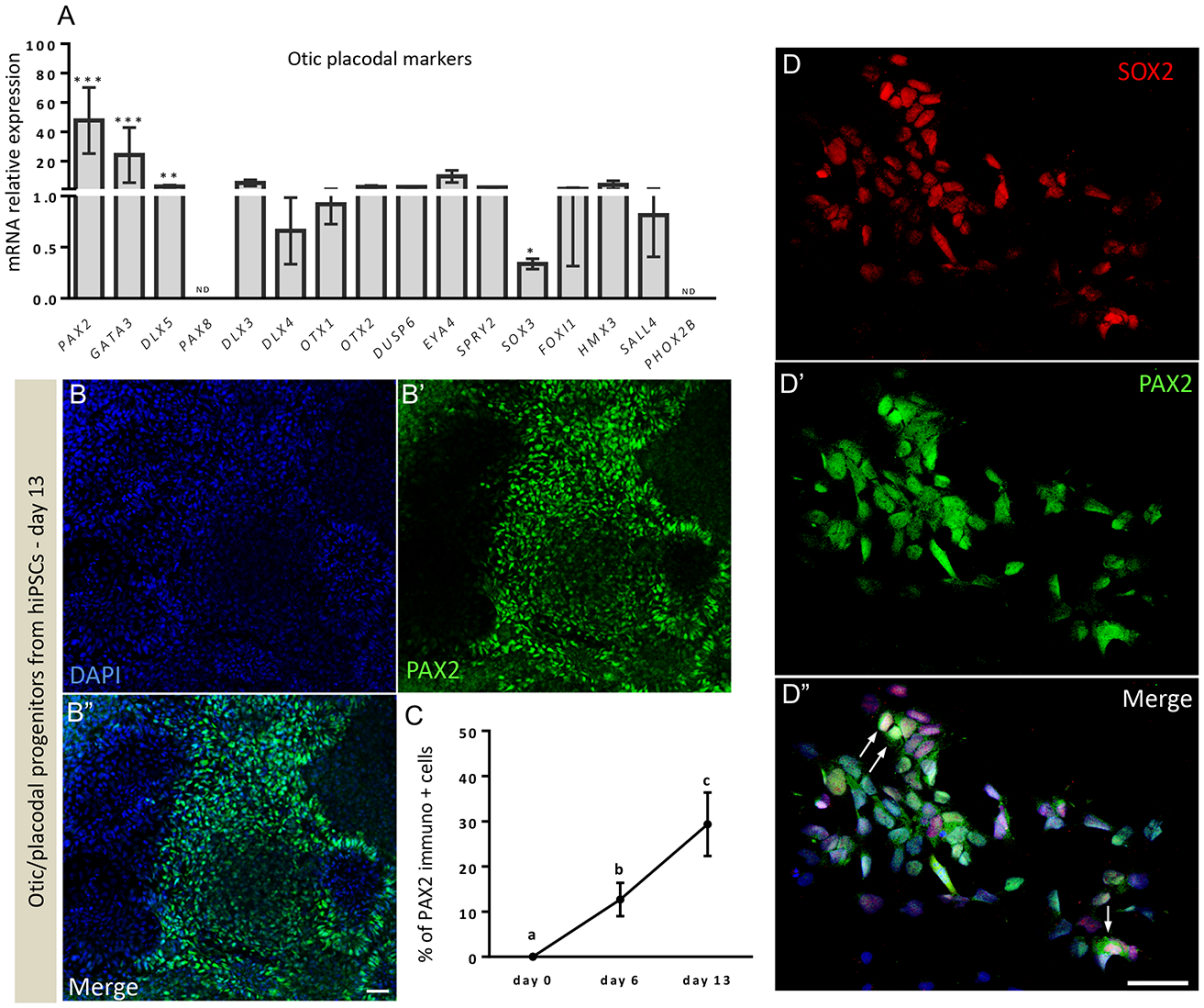
Molecular characterization of otic progenitor cells. (A) RT-qPCR of the expression of several otic/placodal gene markers in FGF3/10 differentiated cells after 13 days *in vitro*. A highly efficient expression of key gene markers (i.e. *PAX2*, *GATA3* and *DLX5*) of otic placodal/lineage was found, while no *PAX8* expression was found at this stage of differentiation. Data were normalized to control for the amount of RNA isolated from undifferentiated hiPSCs and this level of mRNA expression was referred to as 1. Statistical differences were determined with unpaired Student’s t-test. P values are indicated with *p< 0.05, **p < 0.01, and ***p < 0.001 (n = 3 experiments). (B-B”) PAX2 immunoreactivity in differentiated cells further confirmed the otic placodal-like identity at day 13 *in vitro*. (C) Cell quantification revealed a significant increase of PAX2 immuno (+) otic progenitor cells at day 13 when compared to their percentage in cultures at day 6 *in vitro*. Statistical analyses used ANOVA multiple comparisons; different letters (a, b, c) indicate significant differences between groups (at least p < 0.05). (D-D”) Immunocytochemical analyses of the expression of otic/placodal markers (PAX2 and SOX2) in cultures at day 13. A subset of differentiated cells co-expressed SOX2 and PAX2 (arrows), suggesting placode/otic-like identity. Hoechst staining is shown in blue. Scale bars, 50 μm in all panels.

With the aim to study the conversion of hiPSCs to otic/placodal progenitors and their subsequent differentiation into otic sensory lineage, we used a fully characterized hiPSC line expressing the hallmark markers of pluripotency as confirmed by immunolabeling for NANOG, OCT3/4, SSEA4 and SOX2 (S1 Fig). We used a guided strategy with hiPSCs growing as adherent monolayers (Fig 1A–1D). First, in an attempt to derive early otic/placodal progenitors from hiPSCs, we tested whether enriching culture medium with FGF3/FGF10 was sufficient to trigger their induction into early otic lineage, as was previously reported with hESCs [17]. The hiPSCs underwent rapid and profound morphological changes upon FGF3/FGF10 treatment suggesting their differentiation (Figs 1B and 2A). To determine whether the cells had started differentiating toward early otic lineage, we assessed the dynamic expression of a panel of lineage markers known to be expressed during otic/placodal development. We focused on the changes in the expression of transcription factors belonging to the *PAX*, *DLX* and *GATA* gene families, among which some are key markers known to define otic placode identity [10,13]. More specifically, we used a combined expression of *PAX2*, *PAX8*, *GATA3* and *DLX5* gene markers to track the differentiation of hiPSCs towards early otic/placodal lineage. Analysis of transcript relative expression of these genes showed that differentiated cells from hiPSCs started expressing these markers by day 6 *in vitro* (Fig 2B). Confirming these first observations, immunocytochemistry analyses revealed that PAX2, GATA3, DLX5 and PAX8 were also expressed at the protein level in a subset of the differentiated cells (Fig 2C–2F). Interestingly, some of these differentiated cells displayed double immunolabeling for DLX5 and GATA3 markers (Fig 2F). As early as day 6 *in vitro*, quantitative analysis revealed that PAX2, GATA3, DLX5 and PAX8 were respectively expressed by 13.68% ± 2.44%, 10.32% ± 1.57%, 23.03% ± 4.28% and 6.89% ± 4.28% of differentiated cells (Fig 2C). These results suggest that a subset of differentiating cells was rapidly engaged toward otic/placodal cell fate. We then checked whether extending the exposure time to FGF3/FGF10 could contribute towards improving the outcome of otic differentiation (Fig 3A–3C). RT-qPCR analyses of a panel of otic placode markers indicated that *PAX2*, *GATA3* and *DLX5* transcripts significantly increased after 13 days of differentiation, with greater *PAX2* and *GATA3* expression levels compared to those at day 6 (Fig 3A). On the contrary, we observed that *PAX8* expression was lost, confirming the early and transient expression of *PAX8* during otic development. To validate our results, we next quantified PAX2+ cells at day 13 of *in vitro* differentiation (Fig 3B). Interestingly, the temporal analysis of PAX2+ cells showed a significant and progressive increase of PAX2+ cell levels from (12.68% ± 2.12%, **p = 0.04) at day 6 up to 29.34% (± 3.51%, ***p = 0.009) at day 13 (Fig 3C). Of importance, PAX2, a paired box transcription factor, has previously been deemed a crucial otic/placodal lineage marker [39–40].

HMG domain transcription factor, SOX2, is another critical gene for the *in vivo* development of embryonic inner ear. SOX2 expression was found at the earliest stages of development, when the otic placode first appears, and was required for establishment of the progenitor cells within the prosensory domain [41]. We used immunostaining to examine the co-expression of SOX2 and PAX2, to confirm the generation of otic/ placodal progenitors at a cellular level at day 13 (Fig 3D). Immunolabelling showed a subset of SOX2 and PAX2 double positive cells (10% ± 2.18) at day 6 and this cell percentage increased to (15% ± 2.50) at day 13 during the time course of FGF-treated cultures (S2 and S3 Figs).

**Fig 4:**
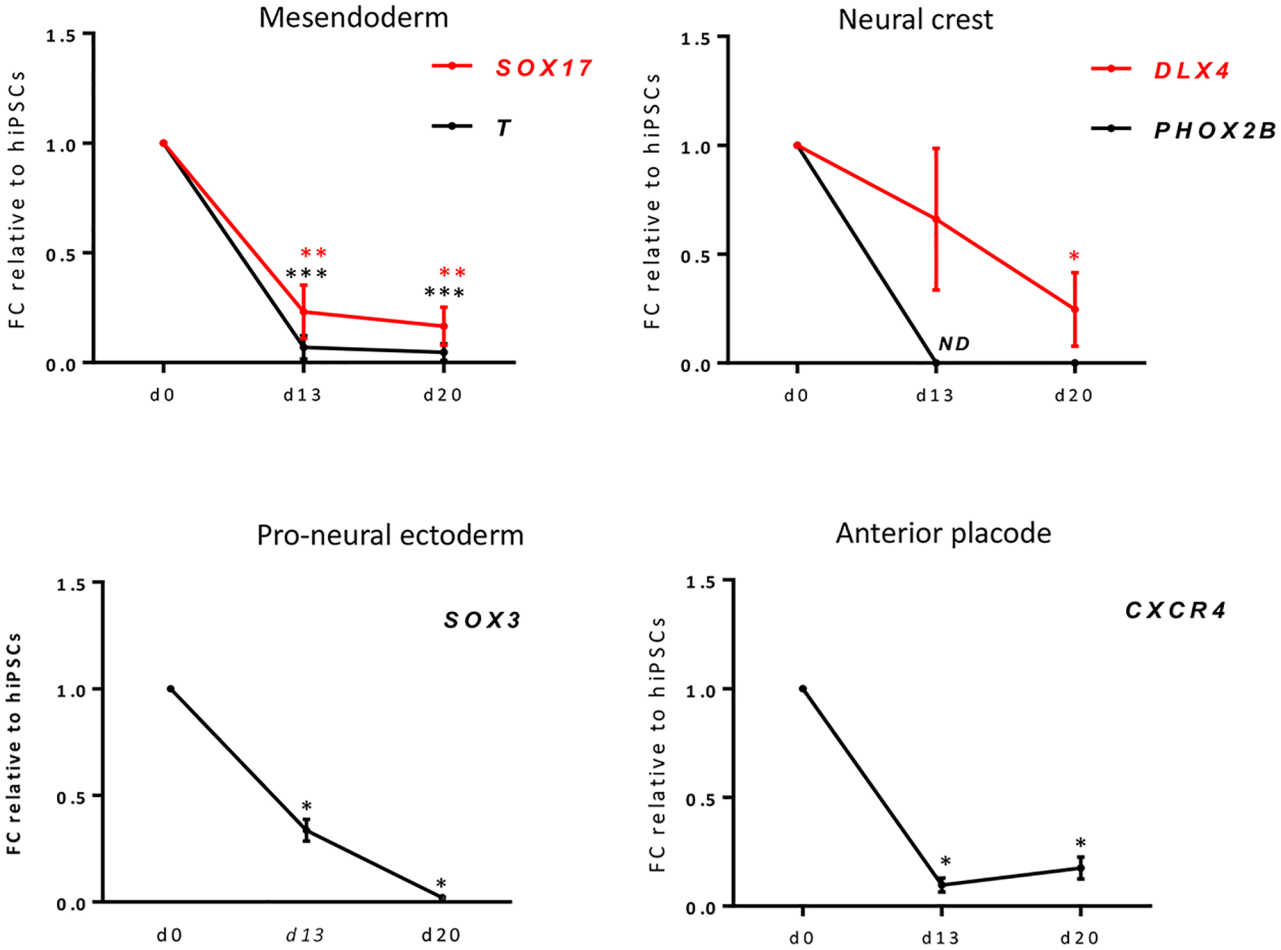
Reduction of meso-endodermal and other lineages during the time course of otic sensory differentiation from hiPSCs. RT-qPCR for changes of *in vitro*-derived cell type markers after 13 and 20 days in comparison to hiPSCs at day 0, normalized to GAPDH gene. Culture conditions are FGF3/10 from d0-d13 followed by DBZ from d13-d20. Expression analyses show downregulation of transcripts for mesoderm (*BRACHYURY*, *T*), endoderm (*SOX17*), neural crest (*DLX4*, *PHOX2B*), pro-neural ectoderm (*SOX3*), and anterior placode (*CXCR4*) lineage markers. Statistical differences were determined with one-way ANOVA. P values are indicated with * for p < 0.05, ** p < 0.001, *** p < 0.0001 in comparison to hiPSCs (n = 3 experiments).

In parallel to the upregulation of otic/placodal markers, there was a decrease in the expression level of known gene markers of mesendoderm and other cell lineages at day 13 *in vitro* (Fig 4). The expression of mesoderm (*Brachyury*, *T*) and endoderm (*SOX17*) genes shown previously to be expressed in hiPSCs [42] had also significantly decreased from d13 and remained low or absent at day 20. We also observed that the pro-neural marker *SOX3* [38], was only weakly detectable at day 13, suggesting a suppressed differentiation of pro-neural ectodermal cells in our guidance strategy. This was associated with a decrease of known neural crest genes including *DLX4* and the homeobox gene *PHOX2B*, considered as essential in establishing neural crest identity [43].

Together, our results demonstrate that hiPSC differentiation in monolayer cultures with FGF3/FGF10 treatment is able to produce presumptive early otic cells that co-express multiple otic/placodal markers as early as day 6 *in vitro*. Extended exposure to the same inducing agents contributed to a significant increase in the number of human otic progenitor-like cells by day 13 *in vitro*.

### Notch inhibition enhances differentiation of otic sensory marker-expressing cells

**Fig 5:**
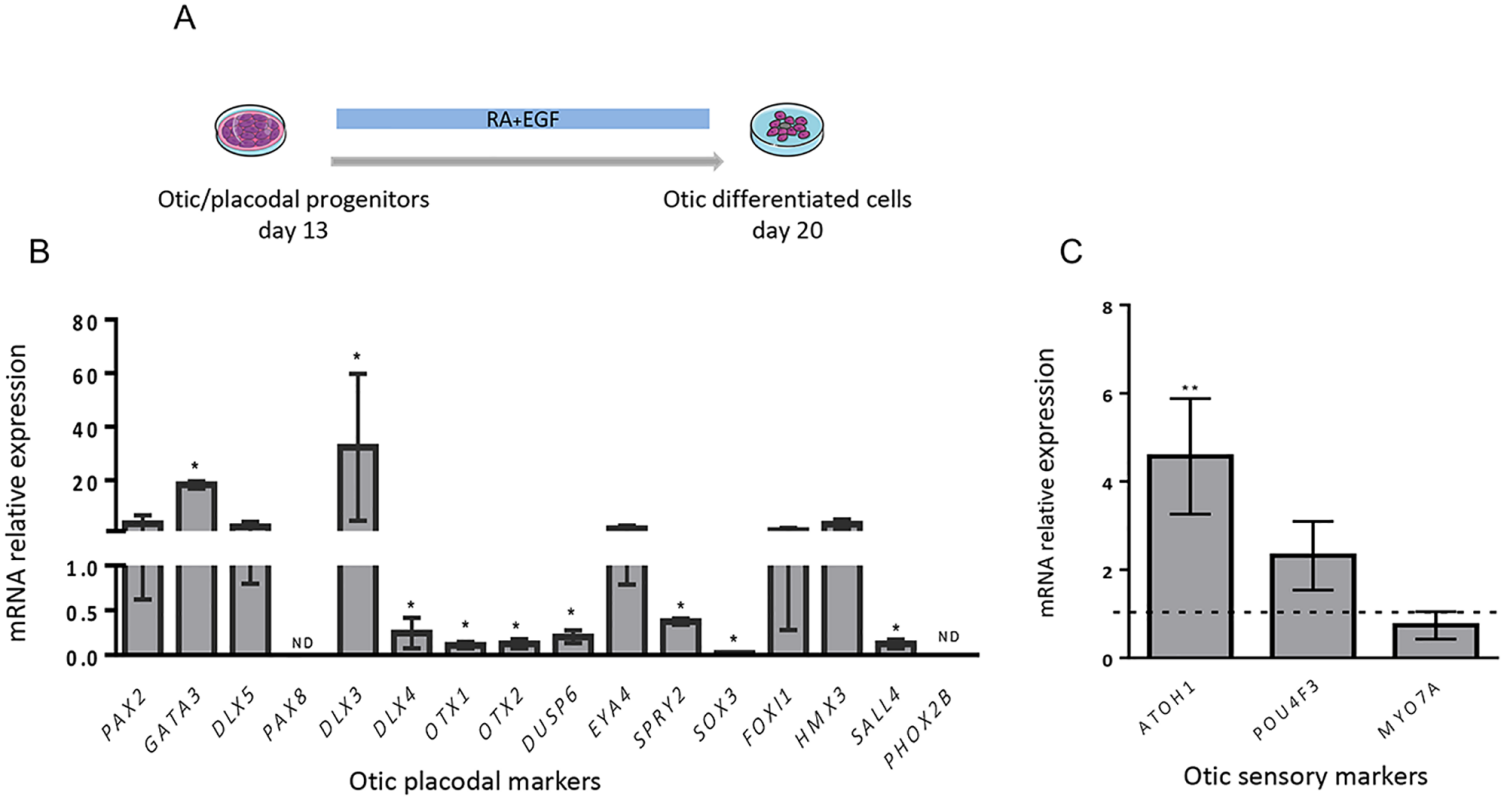
Expression of late otic cell markers. (A) Otic/placodal progenitors from day 13 were maintained for one more week in culture medium supplemented with RA/EGF. (B) The differentiated progenitor cells at day 20 exhibited a significant decrease of a subset of otic/placodal markers as determined by RT-qPCR. (C) In contrast, in these RA/EGF cultures, we observed a notable increase in some gene markers (*ATOH1*, *POU4F3*, *MYO7A*) that define the otic sensory fate with a significant upregulation of *ATOH1* expression. Statistical differences were determined with unpaired Student’s t-test. P values are indicated with *p< 0.05 and **p < 0.01 (n = 3 experiments).

We then wondered whether the newly generated hiPSC-derived otic/placodal progenitors could be further differentiated along the otic sensory lineage to generate cells with initial HC-like phenotype. To this end, progenitor cells at day 13 of differentiation were switched to a culture medium containing RA/EGF, modulators of two pathways that are active during inner ear development. The RA has pleiotropic functions during embryogenesis and has been found to expand otic competence within posterior placode during inner ear development [44–45], while EGF participates towards regulating the *in vitro* production of HCs in embryonic and early postnatal inner ears [46–47]. Of interest, a synergistic interaction between RA and EGF has been found to induce the differentiation of HC-like cells from hESCs [17]. After 7 days differentiation under RA/EGF treatment (Fig 5A), we performed qPCR assays to examine the possible progression of hiPSC-derived otic/placodal progenitors into otic sensory cells. Analysis of transcript expression data revealed a significant downregulation in the expression of a panel of early otic/placodal markers (Fig 5B), suggesting a cell fate transition with decrease of otic/placodal identity and further commitment of the hiPSC-derived otic progenitors. The relative gene expression of *PAX2* returned to basal levels (from 47.67 ± 7.41 at day 13 to 3.79 ± 1.87) at day 20 and was accompanied with a significant upregulation of some initial HC markers, such as *ATOH1* (Fig 5C). The downregulation of pluripotency gene markers confirmed that during *in vitro* differentiation, most cells had lost their pluripotency features by day 20 (S4 Fig). The overall results of these gene expression analyses reveal that in parallel to the loss of early otic placode markers upon RA/EGF treatment, hiPSC-derived otic progenitor-like cells start expressing a subset of otic sensory markers.

In order to assess whether other treatments could promote the differentiation of otic progenitors into cells displaying an otic sensory phenotype, we decided to evaluate the impact of Notch inhibition as compared to RA/EGF treatment in hiPSC cultures. Indeed, previous data from our laboratory and others have demonstrated a crucial role of Notch signaling in otic development and cell-fate decisions [23–28].

**Fig 6:**
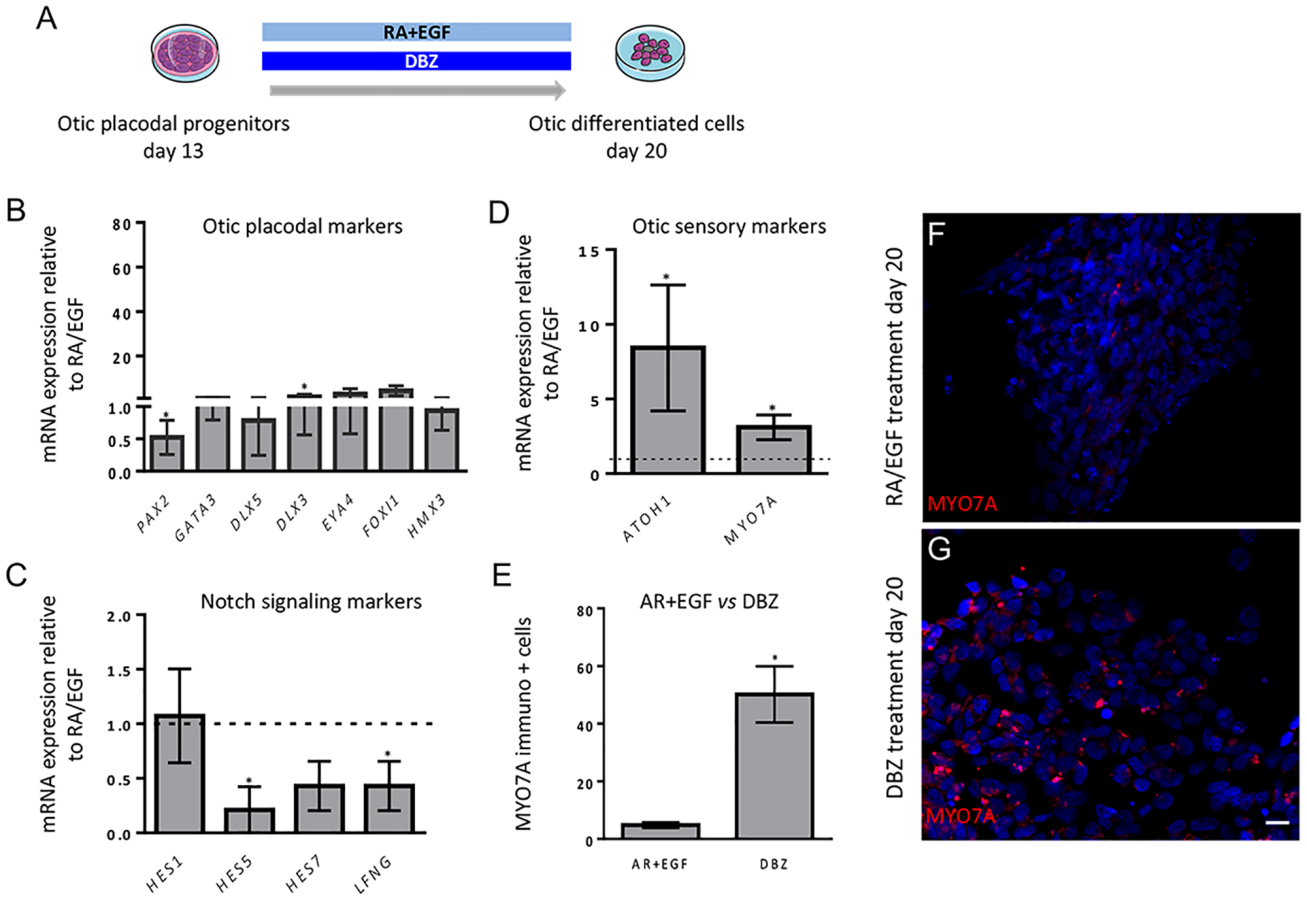
Enhancing the number of human otic sensory cells by Notch inhibition. (A) The otic/placodal progenitors from day 13 were exposed for 7 days (i.e. up to day 20) either to RA/EGF or DBZ compound. (B) Otic/placodal markers were significantly downregulated in DBZ-treated when compared to RA/EGF-treated cells. (C) Downregulation of a subset of Notch signaling genes (*HES5*, *HES7*, *LFNG*) in DBZ-treated when compared to RA/EGF-treated cells. (D) The decrease of Notch pathway components after DBZ treatment was accompanied with a significant upregulation of two otic sensory markers (*ATOH1*, *MYO7A*) transcripts. (E) Quantification of MYO7A+ cells in differentiated cells from DBZ-treated and RA/EGF treated- cultures revealed a much larger fraction of differentiated cells expressing MYO7A (50%*)* in DBZ-treated when compared to RA/EGF-treated cells (4%). The individual bars show the % of immuno(+) cells to the total number of Hoechst-labeled cells. Shown are mean values and standard deviations determined in three independent experiments for each data set. Statistical differences were determined with unpaired Student’s t-test. P values are indicated with * for P < 0.05. (F-G) Representative immunostaining patterns for MYO7A in RA/EGF and DBZ-treated cultures at day 20 *in vitro*. Scale bar, 20 μm.

To evaluate the impact of Notch inhibition on differentiation along the otic sensory lineage *in vitro*, hiPSC-derived otic progenitors from day 13 were subjected to a treatment with gamma-secretase inhibitor (DBZ compound) over a one-week period (Fig 6A). At day 20, differentiated cells were harvested to analyze and compare the expression levels of otic sensory markers between DBZ and RA/EGF-treated age-matched cultures (Fig 6B–6E). Similarly to what we observed after RA/EGF treatment, qPCR assays on DBZ-treated cells showed a decrease in expression levels of a subset of otic/placodal markers. Remarkably, *PAX2* downregulation was significantly greater in DBZ compared to RA/EGF-treated cultures.

In the culture condition in which hiPSCs were differentiated in DFNB medium, the relative expression of early otic/placodal was very low for *PAX2* at day 13 and increased for *GATA3*, *DLX3/5* at day 20. Of interest, the relative expression levels of the late otic markers *ATHO1* and *MYO7A* remained undetectable during the time course of *in vitro* differentiation in DFNB culture medium (S4 Fig).

To examine the role of Notch signaling in differentiation along the otic sensory lineage, we then evaluated the gene expression levels of major downstream effectors of the Notch pathway with known involvement in cell fate within the developing inner ear [48–50]. Our qPCR analyses revealed that DBZ treatment leads to a significant downregulation in expression of *HES5* and *LFNG*, two crucial components of the Notch pathway (Fig 6C). This decrease was accompanied by a significantly greater increase in the expression of the initial HC marker *ATOH1* in DBZ as compared to RA/EGF-treated cultures, suggesting a greater effect of Notch inhibition on otic sensory commitment. In addition, we observed a significant upregulation of *MYO7A* in DBZ-treated cultures (Fig 6D). MYO7A being one of the specific initial HC proteins, we next assessed MYO7A protein expression in the DBZ-treated cells. Immunocytochemistry analysis confirmed the expression of MYO7A in DBZ-treated cultures. Strikingly, around half (50.15% ± 9.75%, *p = 0.04) of the differentiated cells were immunopositive for MYO7A following DBZ treatment (Fig 6E–6G) in contrast to only 4.83% (**±** 0.96%) of those following RA/EGF treatment (Fig 6E and 6F).

**Fig 7:**
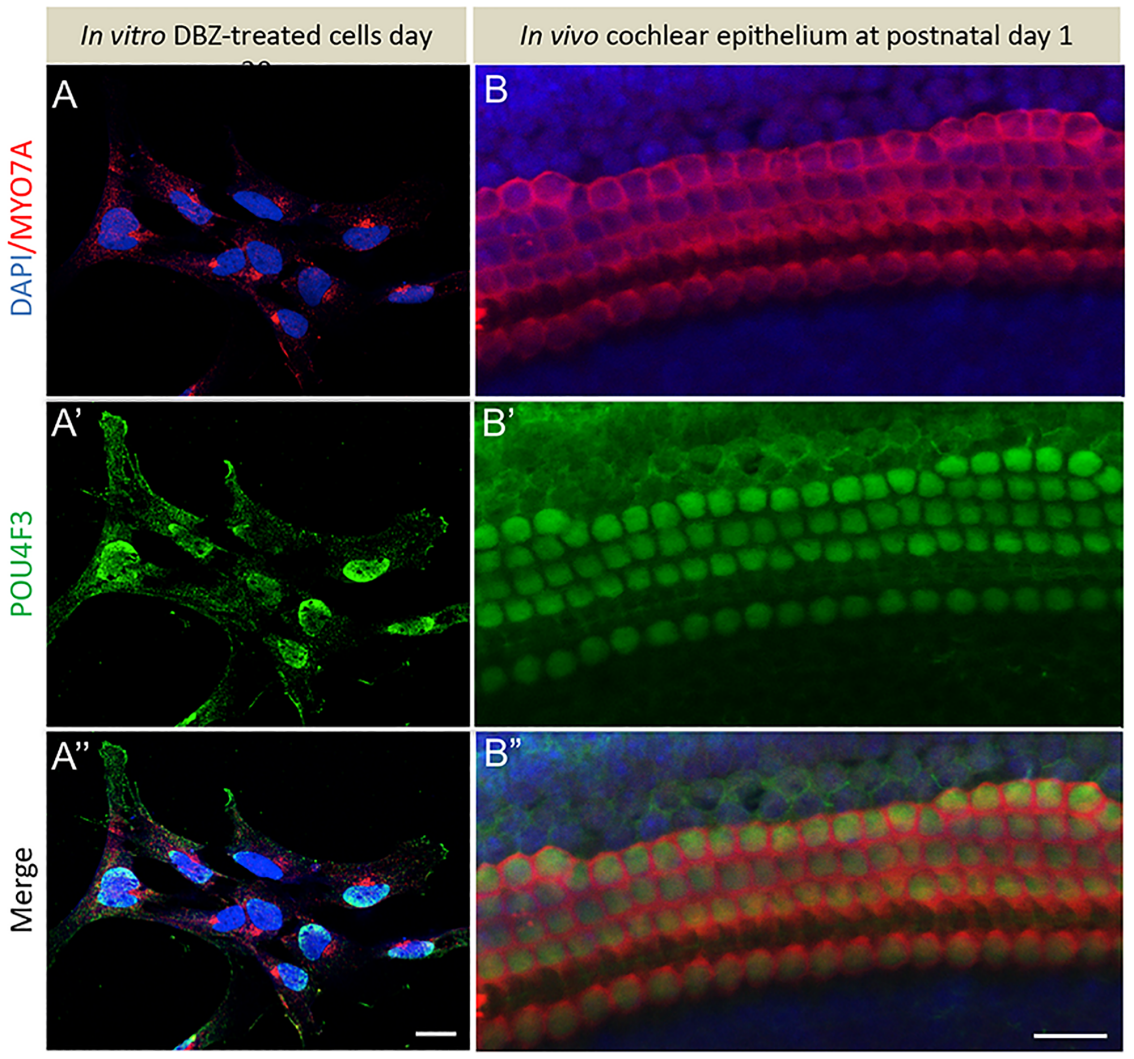
Expression pattern of otic sensory markers *in vitro* mimics that in early developing cochlear epithelium. (A-A”) After one week of treatment with DBZ, a subset of differentiated cells displayed co-expression MYO7A in the cytoplasm (shown in red) and POU4F3 in the nucleus (shown in green). (B-B”) The expression pattern of MYO7A and POU4F3 in *in vivo* mouse cochlear whole-mount preparations. Scale bars, 20 μm.

Furthermore, co-immunostaining analysis revealed that MYO7A+ cells co-expressed another initial HC marker i.e., POU4F3. Of importance, the specificity of the MYO7A and POU4F3 immunophenotype in DBZ-differentiated cells (Fig 7A–7A”) was corroborated by their expression in whole-mount preparations of postnatal day 1 mouse cochlea (Fig 7B–7B”). In addition, we found the same expression pattern to that *in vivo*, with POU4F3 confined to the nucleus and MYO7A located in the cytoplasm. Noticeably, the expression of MYO7A in the differentiated cells was mainly confined in a perinuclear cap-like structure, perhaps indicating a polarization of the generated cells (Fig 7A”) in line with a HC-like phenotype, though it may also reflect their immaturity when compared to their *in vivo* counterparts showing broader distribution within the entire cytoplasm.

We also tested for the expression of genes characteristic of other epithelial cells in the inner ear such as supporting cells. There was evidence that at least some supporting cell differentiation was taking place under our culture conditions. In the differentiated cells obtained at day 13 and day 20 *in vitro*, genes characteristic of supporting cells (*S100A1*, *JAG1*, *SOX2*, *GJB1*, and *LGR5*) were also observed (S5 Fig).

Moreover, GJB1, encoding CONNEXIN 32 and forming part of the gap junction between supporting cells *in vivo*, was highly detected at day 20 after exposition of progenitor cells to DBZ compound as compared to other conditions. The SOX2 is detected at day 20, however, its expression is prominent in FGF3/10 cultures at day 13, consistent its dual role in early otic placode specification and supporting cell determination during inner ear development. In addition, we detected a population of SOX2 immuno+ cells intermixed with differentiated cells that became Myosin7A immuno+ cells at day 20 *in vitro* (S6 Fig).

Altogether our data suggest that Notch inhibition promotes the differentiation of hiPSC-derived otic/placodal to an enriched set of cells that had expression profiles characteristic of otic sensory cells *in vitro*.

## Discussion

Regenerative medicine offers reasonable expectations for the potential treatment of inner ear disorders through the replacement of lost or damaged sensory cells. Initial advances in the differentiation of murine ESCs/iPSCs into HC-like cells [9–15] have paved the way for similar progress with pluripotent stem cells of human origin. Compelling evidence accumulated over the last decade supports hiPSC technology as offering a promising future for stem cell research, disease modeling and cell-based therapies in different types of tissues. In the case of the inner ear, one of the challenges is to better define the developmental pathways and their sequential activation/inactivation to allow the efficient *in vitro* production of human inner ear otic/placodal progenitors and their further differentiation into otic sensory cell-like cells. In the present study, our aim was to promote human otic/placodal induction processes and the generation of otic sensory lineage cells by exploring the effects of timely modulations of major pathways during hiPSC differentiation. Our results show that the expression of otic/placodal markers can be induced through the activation of the FGF signaling pathway by FGF3 and FGF10 ligands, suggesting that human placodal development and otic induction from hiPSCs is also an FGF-dependent process, as previously demonstrated with lineage guidance of both mESCs [9–14] and hESCs [17–20]. FGF activation has emerged as a prominent player in promoting otic-epibranchial progenitor identity during early development [51–52]. Indeed, ectopic expression of FGF3 or FGF10 during mouse embryogenesis induces the formation of ectopic otic vesicles expressing some otic markers i.e., *PAX2* [53]. Complementary loss-of-function approaches in zebrafish revealed that high levels of *PAX2A* and *PAX8* favor otic differentiation, whereas low levels increase cell numbers in epibranchial ganglia [54]. In addition, previous data reported that FGF signaling is important at early stages to induce expression of *PAX2* and *PAX8* required for otic induction through differential regulation of competence factors *FOXI1*, *SOX3* and *FGF24* [55]. Interestingly, in our gene expression analysis of differentiated cells at day 13, we found a significant upregulation of *PAX2* in parallel to a downregulation of *SOX3*, known as a pro-neural ectoderm lineage marker. In addition to *PAX2*/*PAX8* expression, our results showed a population of hiPSC-derived otic/placodal progenitors that co-expressed other markers, such as *DLX5* and *GATA3* which are generally found in the native otic placode. While not specific individually, the combined expression of multiple otic/placodal gene markers is thought to be a good indicator of otic lineage identity [10–13]. Our results suggest that FGF treatment of hiPSCs in monolayer cultures promotes human otic/placodal progenitors while reducing or suppressing mesendoderm and pro-neural ectoderm lineages. The generation of different otic/placodal lineages implies the homogeneous nature of differentiation allowed by adherent monolayer system [19, 56] compared to EB-aggregates or 3D-based culture strategies [12, 57]. In the second step of the procedure, we tested which *in vitro* conditions would enhance the ability of hiPSC-derived otic progenitors to differentiate into human otic sensory cells. To this end, we explored the differentiation potential of otic progenitor cells under Notch inhibition (i.e. DBZ-treated) as compared to RA/EGF treatment previously used to induce HC-like cells *in vitro* from hESCs [17]. We used multiple initial HC gene markers (*ATOH1*, *POU4F3* and *MYO7A*) to examine a possible sensory cell identity after challenging human otic progenitors with either DBZ compound or RA/EGF supplements for an additional 7 days in culture. The otic sensory lineage specification depends on the proneural gene *ATOH1* and its interactions with other transcription factors [58]. *ATOH1* is necessary and in some contexts sufficient for early inner ear HC development [59]. The *POU4F3* and *MYO7A* have been found expressed in differentiating HCs during early inner ear development [57–58] shortly after *ATOH1* and are considered to be among the crucial initial HC markers in otic sensory lineage studies [29, 59]. Of interest, our qPCR analysis revealed a significant upregulation of *ATOH1* expression when early otic progenitor cells were exposed to RA/EGF. This observation fits with the roles of EGF and retinoid pathways in inner ear development and with a previous study on hESCs [17]. RA has also been shown to regulate otic vesicle formation *in vivo* [44–45], whereas EGF ligands i.e., EGF/TGF-alpha promote proliferation and/or maintenance of inner ear progenitor cells *in vitro* [46–47]. In contrast, for otic progenitors maintained 7 days *in vitro* under Notch inhibition, we observed a significant increase in the relative gene expression of both *ATOTH1* and *MYO7A* as compared to their levels in age-matched RA/EGF-treated cultures. In addition, challenging otic progenitors with DBZ resulted in the generation of half (_~_50% of total) being MYO7-immunopositive compared to around 5% within the RA/EGF-treated cultures. Our immunostaining results provide additional insight and support the efficient promotion of otic sensory lineage achieved under Notch modulation, which led to the differentiation of sensory cell populations that were double immuno+ for MYO7A and POU4F3. Interestingly, using an embryoid body model, Costa et al. [13] generated HC-like cells (i.e., MYO7A immuno+) using mESCs by genetic programming through combined overexpression of *ATOH1*, *GFI1* and *POU4F3* transcription factors. Interestingly, compared to RA/EGF cultures, DBZ cultures showed a concomitant decrease in the expression of the bHLH gene *HES5* and the Fringe gene *LNFG* both of which are components of the Notch pathway. This result is in accordance with our previous observation of a significant downregulation of *HES5* following pharmacological inhibition of Notch in mouse inner ear tissue specific-stem cells differentiated in a sphere model [60]. Furthermore, a recent study demonstrated that initial pro-sensory cell fate is regulated by Fringe activity, requires low levels of Notch signaling and is sensitive to changes in Notch signaling in the developing organ of Corti [28]. Another subsequent cell fate decision operates via a lateral inhibition mechanism to sort out HCs and supporting cells during inner ear development [23^_^^25^]. This second cell fate process was reported as independent of Fringe activity and much less sensitive to small changes in Notch activity [28]. Taking into account the increase in the expression of otic sensory markers and downregulation of *HES5* and *LNFG* known to modify Notch signal transduction properties [61], it is reasonable to presume that in our *in vitro* differentiation system, a pharmacological reduction of Notch activity would affect the Fringe-dependent otic sensory cell fate process. Our results revealed that hiPSC-derived otic progenitors were capable of differentiation into cells expressing markers for otic sensory lineage. This differentiation ability towards otic sensory lineage was enhanced by a pharmacological modulation of Notch pathway *in vitro*. Although, human otic progenitors differentiated either under EGF/RA or DBZ expressed initial HC markers MYO7*A* and POU4F3, their expression was not sufficient to promote the formation of stereocilia, as reported with inner ear organoids [20]. However, the 3D culture protocol used to generate functional HC-like cells from hESCs in this previous study involved multiple steps and was time-consuming (up to 75 days) as compared to the monolayer culture system used the present study (20 days). In addition, mature HCs differentiated in organoid aggregates may not be appropriate for further cell transplantation experiments that require hydrogel-free culture of differentiated cell progenitors. The lack of hair bundle-like structures in our hiPSC culture system suggested that human otic sensory cells were at a nascent state of commitment to HC phenotype and had failed to continue final maturation.

**Fig 8:**
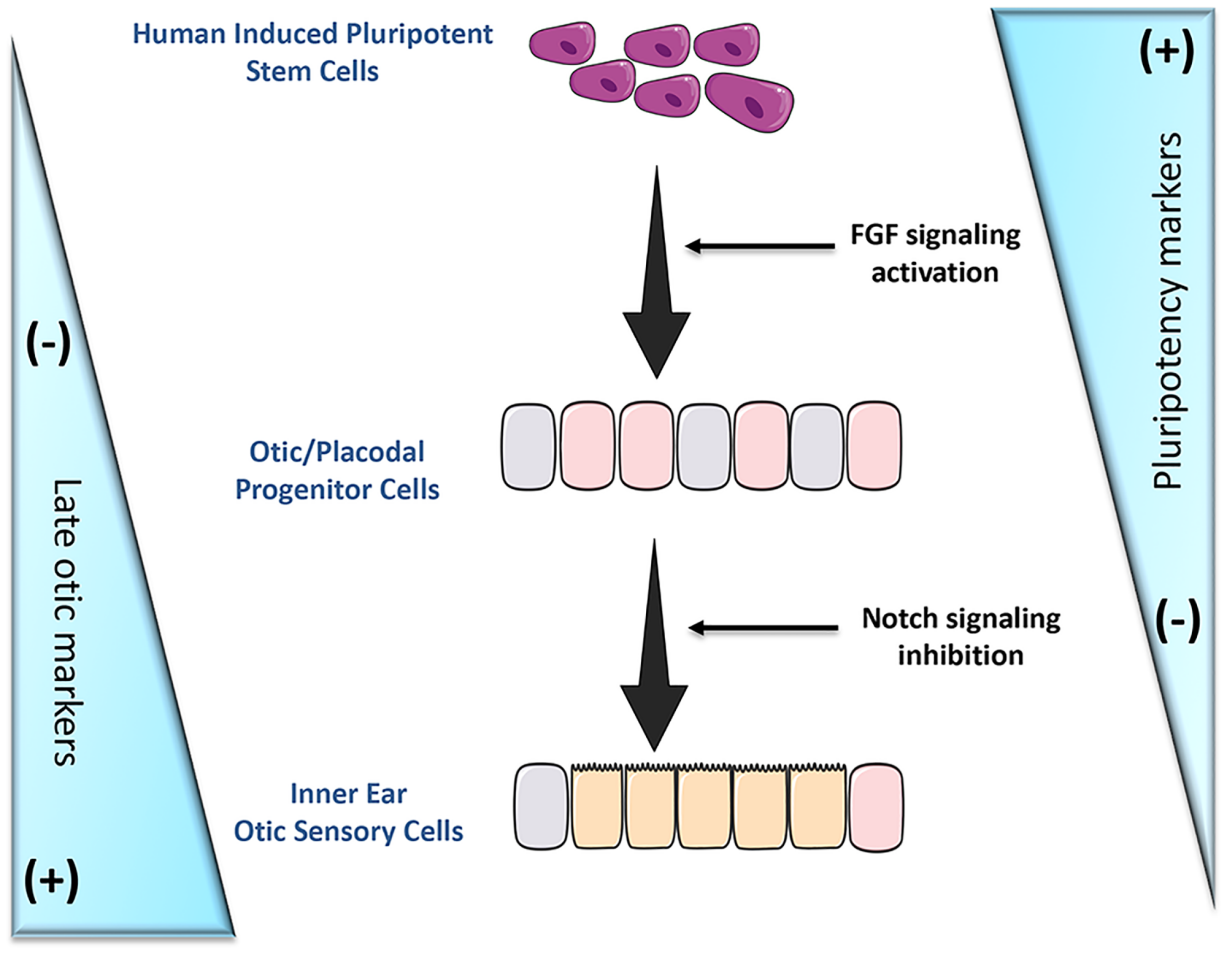
Schematic representation of the generation of inner ear otic sensory cells from hiPSCs by dual stepwise activation of FGF and inhibition of Notch signaling pathways in adherent monolayer cultures.

However, once improved, the *in vitro* differentiation procedure used in the present study might provide a simple and rapid approach to generating human inner ear HC-like cells harboring functional hair bundles. In conclusion, our findings provide a useful human induced pluripotent stem cell differentiation assay to generate large numbers of otic/placodal progenitors with a propensity to give rise to otic sensory cells (Fig 8). This study could provide the foundations on which to build a more solid understanding of the mechanisms controlling otic sensory differentiation and represents a useful step towards future explorations of the transplantation potential of characterized human otic progenitors.

## Supporting information

S1 Fig Characterization of pluripotency markers of cultured hiPSCs. Immunostaining of undifferentiated hiPSCs with antibodies specific for pluripotency markers: NANOG, SSEA4, OCT4, SOX2, and AP activity. (A-C) The pluripotency marker molecules were expressed in virtually all the propagated cells. The immunostaining also revealed the lack of the early differentiation marker SSEA1 in hiPSCs maintained in DEF-CSTM 500 culture medium. These immunostainings are representative for three hiPSC propagation experiments. Nuclei were stained with Hoescht (blue). Scale bars, 50 μm. Abbreviations: hiPSCs, human induced pluripotent stem cells; AP, alkaline phosphatase.
(TIF)

S2 Fig Generation of otic/placodal cells from hiPSCs cultures at day 13 of in vitro differentiation. Representative double immunostainings for PAX2 and SOX2 (A-A”) and for PAX2 and DLX5 (B-B”) in FGF3/10 cell cultures. A population of PAX2 and SOX2 double immuno+ cells (dotted circle) are observed in these differentiated cultures. In some areas within the colonies, cells co-expressed DLX5 and PAX2 (arrows). Hoechst staining is shown in blue. Scale bars, 20 μm (A-A”); 50 μm (B-B”).
(TIF)

S3 Fig Quantification of otic/placodal labeled cell expression of differentiated cells in FGF3/10 cultures at day 6 and day 13 in vitro. The individual bars visualize the fraction of positive immunolabelled cells to the total number of Hoechst labeled-cells examined in eleven randomly selected distinct fields from five coverslips (n = 1).
(TIF)

S4 Fig Analysis of pluripotency and otic gene markers by RT-QPCR during the time course of hiPSC differentiation. (A) A progressive downregulation in the relative gene expression of a subset of pluripotency factors during differentiation processes following exposition to FGF3/10 and RA/EGF at day 13 (B) and day 20 (C) cultures respectively. (D) Expression of early otic/placodal and late otic markers at day 13 and day 20 of *in vitro* differentiation in DFNB medium alone. Note the increase in the relative expression of *GATA3*, *DLX3/5* at day 20 and a very low expression level of *PAX2* at day 13 and day 20. For late otic markers (i.e. *ATHO1* and *MYO7A*) their expression levels remained undetectable during the time course of differentiation in DFNB medium. Statistical differences were determined with unpaired Student’s t-test (n = 3 experiments for B, C). Significant differences are indicated by *p< 0.05. For D, one experiment with 2 biological duplicates/ culture condition.
(TIF)

S5 Fig A gene expression panel of six known supporting cell markers during the time course of hiPSC differentiation. RT-qPCR for changes of supporting cell markers at day 13 and day 20 of differentiation in comparison to undifferentiated cells at day 0, normalized to GAPDH gene. Expression analyses show increase in transcripts of S100A1, LGR5, JAG1, HEY1 and SOX2 in FGF3/10 cultures. After exposition to DBZ (1–5 μM), we noticed an increase in the expression of GJB1 and a decrease of HEY1 transcripts. Statistical differences were determined with unpaired Student’s t-test (n = 3 experiments).
(TIF)

S6 Fig Otic sensory cell cultures previously exposed to DBZ were maintained for one additional week (day-27) in DFNB culture medium. These differentiated cultures displayed MYO7A + cells (shown in red) intermixed with a large population of SOX2 expressing cells (shown in green) (A-A’). Scale bar = 50 μm.
(TIF)

S1 Table List of gene-specific primers used for RT-qPCR for gene expression studies. (DOCX)

S2 Table List of antibodies used in immunocytochemical marker expression studies. (DOCX)
