# Supplementary figures and images for: Modeling human early otic sensory cell development with induced pluripotent stem cells
Source: PLoS One. 2018 Jun 14;13(6):e0198954. doi: 10.1371/journal.pone.0198954 (PMC6002076; doi:10.1371/journal.pone.0198954)

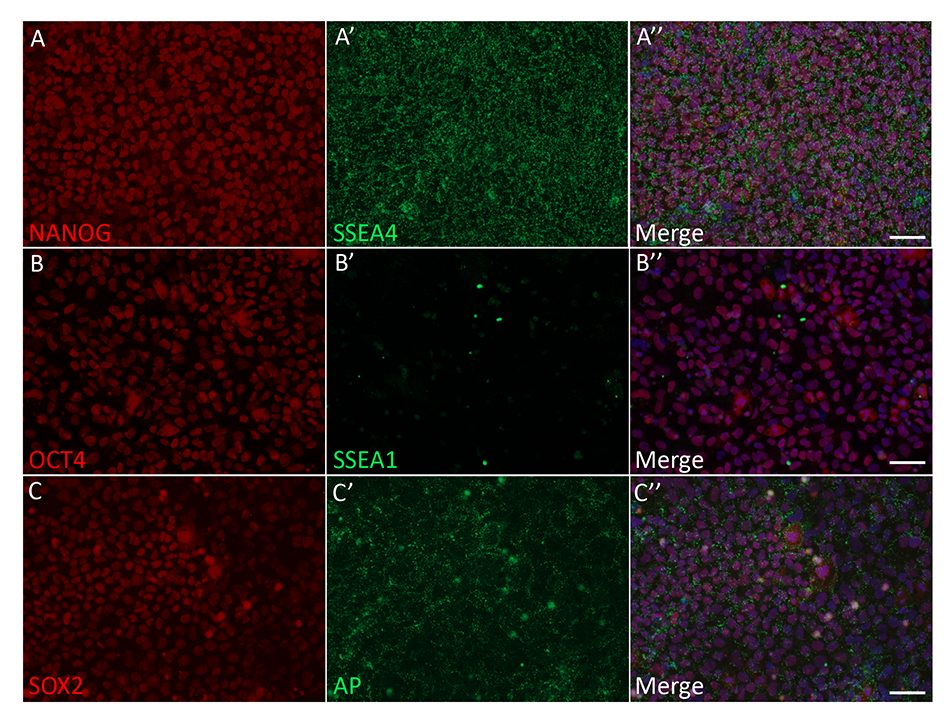

Supplement: S1 Fig — Immunostaining of undifferentiated hiPSCs with antibodies specific for pluripotency markers: NANOG, SSEA4, OCT4, SOX2, and AP activity. (A-C) The pluripotency marker molecules were expressed in virtually all the propagated cells. The immunostaining also revealed the lack of the early differentiation marker SSEA1 in hiPSCs maintained in DEF-CSTM 500 culture medium. These immunostainings are representative for three hiPSC propagation experiments. Nuclei were stained with Hoescht (blue). Scale bars, 50 μm. Abbreviations: hiPSCs, human induced pluripotent stem cells; AP, alkaline phosphatase. (TIF) [file pone.0198954.s001.tif]

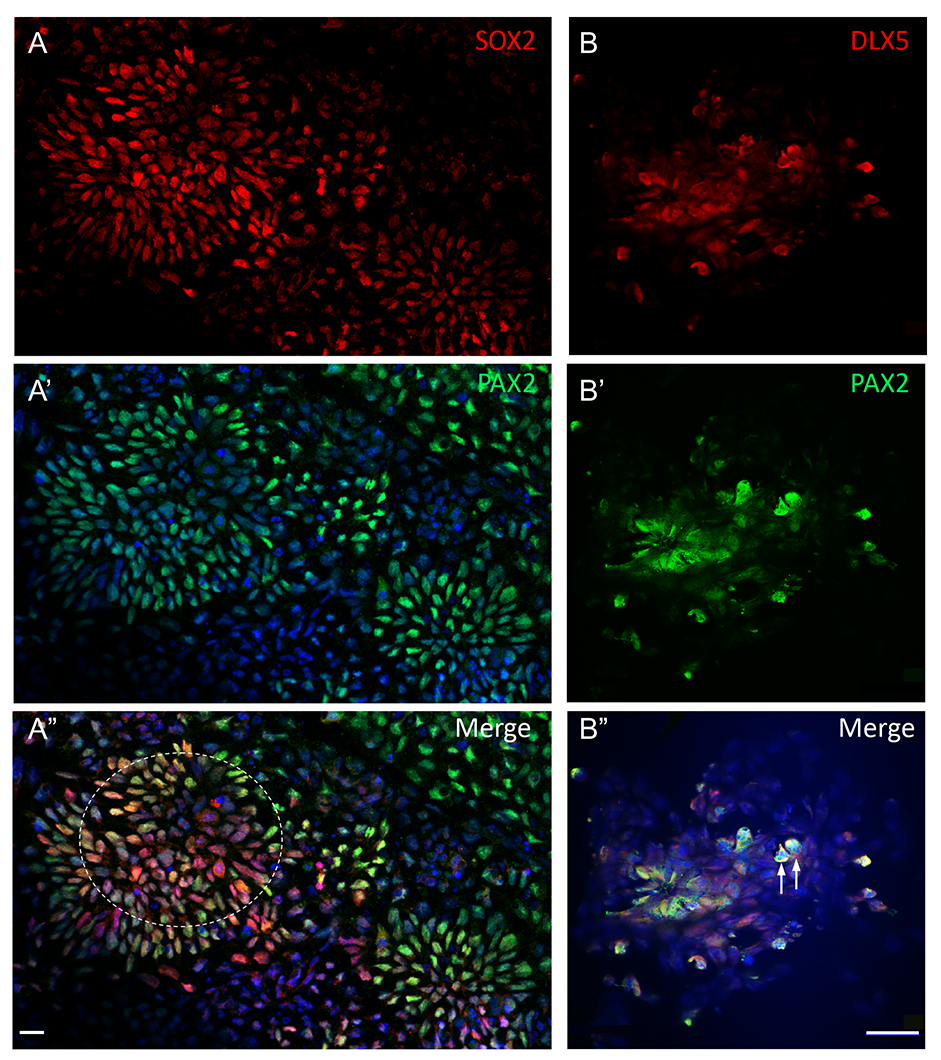

Supplement: S2 Fig — Representative double immunostainings for PAX2 and SOX2 (A-A”) and for PAX2 and DLX5 (B-B”) in FGF3/10 cell cultures. A population of PAX2 and SOX2 double immuno+ cells (dotted circle) are observed in these differentiated cultures. In some areas within the colonies, cells co-expressed DLX5 and PAX2 (arrows). Hoechst staining is shown in blue. Scale bars, 20 μm (A-A”); 50 μm (B-B”). (TIF) [file pone.0198954.s002.tif]

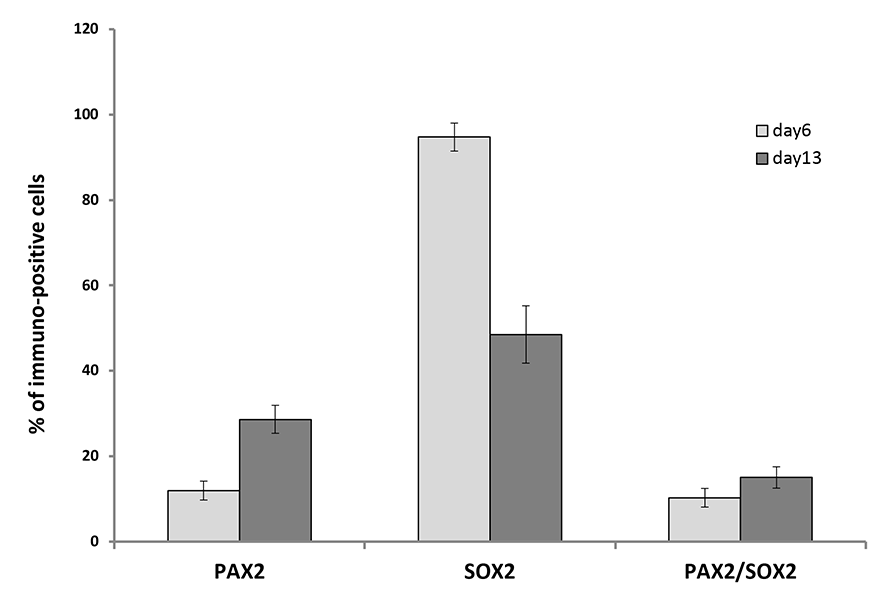

Supplement: S3 Fig — The individual bars visualize the fraction of positive immunolabelled cells to the total number of Hoechst labeled-cells examined in eleven randomly selected distinct fields from five coverslips (n = 1). (TIF) [file pone.0198954.s003.tif]

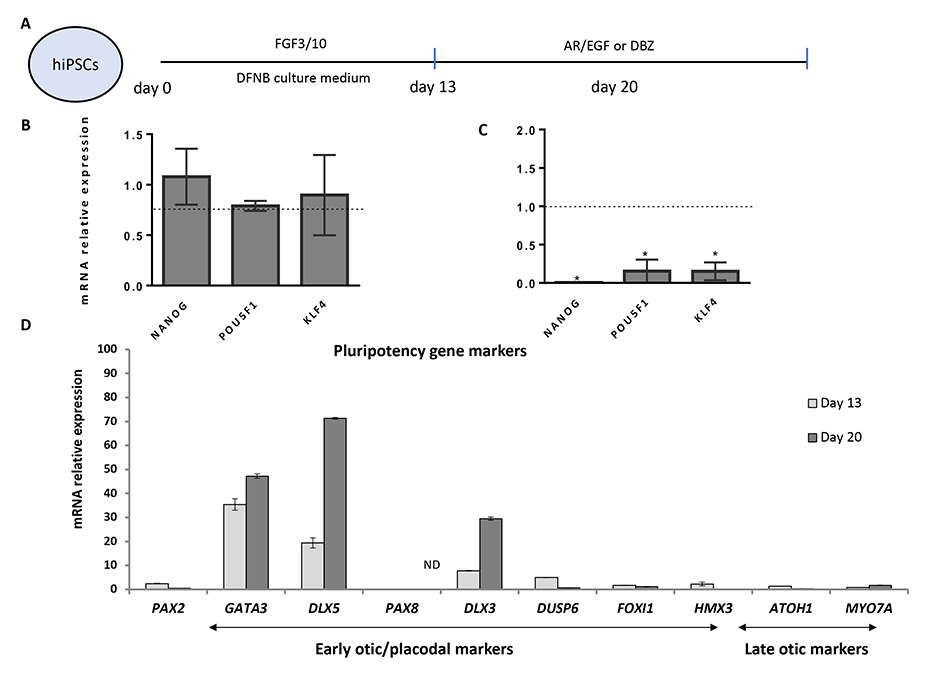

Supplement: S4 Fig — (A) A progressive downregulation in the relative gene expression of a subset of pluripotency factors during differentiation processes following exposition to FGF3/10 and RA/EGF at day 13 (B) and day 20 (C) cultures respectively. (D) Expression of early otic/placodal and late otic markers at day 13 and day 20 of in vitro differentiation in DFNB medium alone. Note the increase in the relative expression of GATA3, DLX3/5 at day 20 and a very low expression level of PAX2 at day 13 and day 20. For late otic markers (i.e. ATHO1 and MYO7A) their expression levels remained undetectable during the time course of differentiation in DFNB medium. Statistical differences were determined with unpaired Student’s t-test (n = 3 experiments for B, C). Significant differences are indicated by *p< 0.05. For D, one experiment with 2 biological duplicates/ culture condition. (TIF) [file pone.0198954.s004.tif]

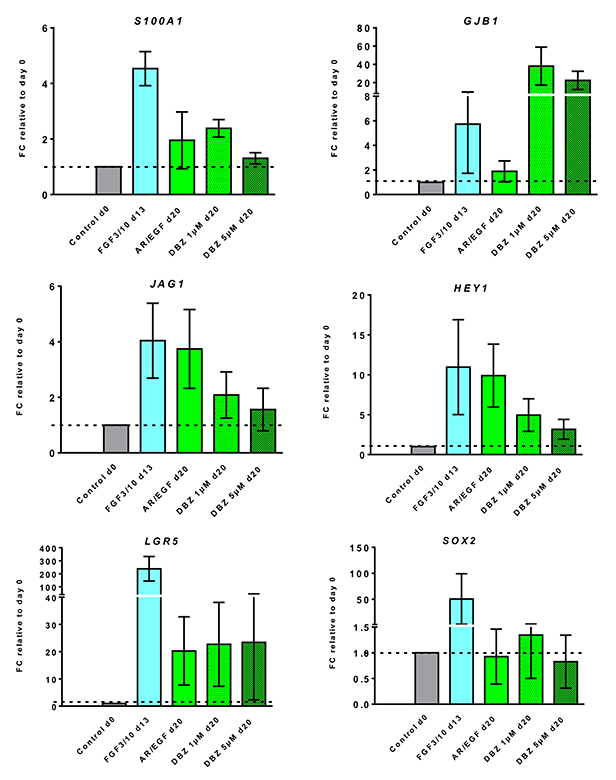

Supplement: S5 Fig — RT-qPCR for changes of supporting cell markers at day 13 and day 20 of differentiation in comparison to undifferentiated cells at day 0, normalized to GAPDH gene. Expression analyses show increase in transcripts of S100A1, LGR5, JAG1, HEY1 and SOX2 in FGF3/10 cultures. After exposition to DBZ (1–5 μM), we noticed an increase in the expression of GJB1 and a decrease of HEY1 transcripts. Statistical differences were determined with unpaired Student’s t-test (n = 3 experiments). (TIF) [file pone.0198954.s005.tif]

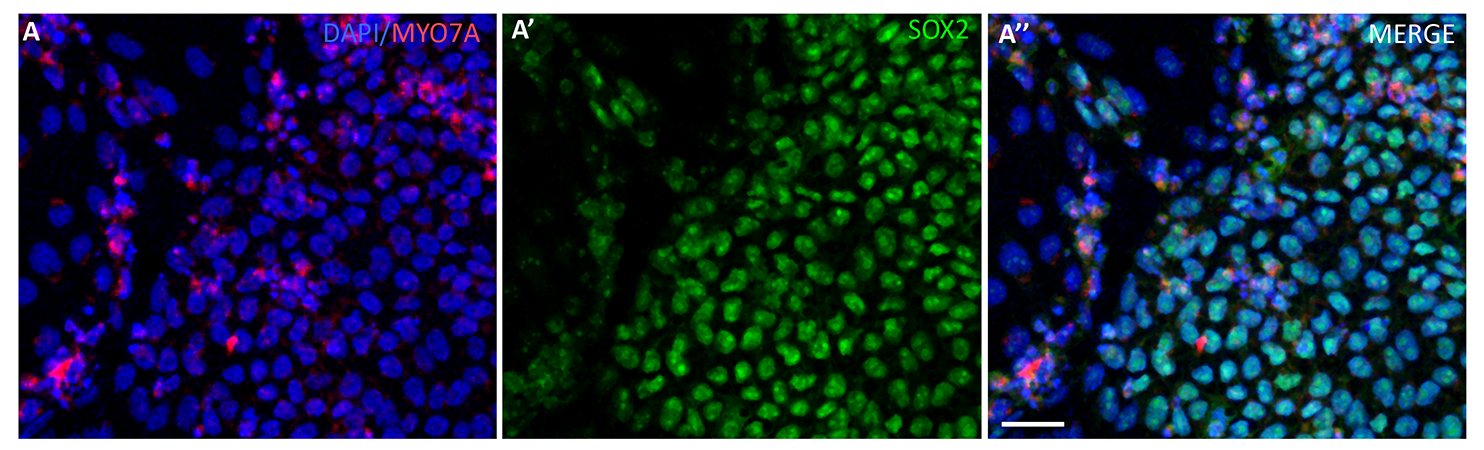

Supplement: S6 Fig — These differentiated cultures displayed MYO7A + cells (shown in red) intermixed with a large population of SOX2 expressing cells (shown in green) (A-A’). Scale bar = 50 μm. (TIF) [file pone.0198954.s006.tif]
