# Supplementary material for: Modeling human early otic sensory cell development with induced pluripotent stem cells
Source: PLoS One. 2018 Jun 14;13(6):e0198954. doi: 10.1371/journal.pone.0198954 (PMC6002076; doi:10.1371/journal.pone.0198954)
Supplement: S1 Table — (DOCX) [file pone.0198954.s007.docx]

|  | **Species** | **Provider** | **Reference** | **Dilution** |
| --- | --- | --- | --- | --- |
| **Alkaline phosphatase** | mouse | Life Technologies | Part#962647 | 1 : 100 |
| **DLX5** | goat | Santa-Cruz | SC-18152 | 1 : 50 |
| **GATA3** | mouse | Santa-Cruz | SC-268 | 1 : 50 |
| **MYO7A** | rabbit | Proteus (Coger) | PTS-25-6790-C050 | 1 : 200 |
| **NANOG** | goat | Life Technologies | Part#963488 | 1 : 100 |
| **OCT3/4** | goat | Life Technologies | Part#962649 | 1 : 100 |
| **PAX2** | rabbit | Covance | PRB-276P | 1 : 100 |
| **PAX8** | goat | Covance | SC-16279 | 1 : 100 |
| **POU4F3** | mouse | Abnova (Interchem) | H-5459-M01(DB9310) | 1 : 100 |
| **Sox2** | goat | Santa-Cruz | SC-17320 | 1 : 200 |
| **Sox2** | rabbit | Millipore | AB5603 | 1 : 200 |
| **SSEA1** | mouse | Life Technologies | Part#963489 | 1 : 100 |
| **SSEA4** | mouse | Life Technologies | Part#962648 | 1 : 100 |
