# Supplementary material for: Modeling human early otic sensory cell development with induced pluripotent stem cells
Source: PLoS One. 2018 Jun 14;13(6):e0198954. doi: 10.1371/journal.pone.0198954 (PMC6002076; doi:10.1371/journal.pone.0198954)
Supplement: S2 Table — (DOCX) [file pone.0198954.s008.docx]

| **Gene Name** | **Forward Primer** | **Rerverse Primer** |
| --- | --- | --- |
| *ATOH1* | GCAATGTTATCCCGTCGTTCAA | TCGGACAAGGCGTTGATGTA |
| *DLX3* | AGCCTCCTACCGGCAATAC | TTCCGGCTCCTCCTTCAC |
| *DLX4* | GTTTCCAGCACACGCAGTAC | GCGTTTGTTCTGAAACCAGATCTT |
| *DLX5* | GCTAGCTCCTACCACCAGTAC | GGTTTGCCATTCACCATTCTCA |
| *DUSP6* | AGCAGCCCATGTGACAACA | TGCAGAGAGTCCACCTGGTA |
| *EYA4* | CCCTTGAACAGCAGTGAAACC | GGGCTGTAGCCACTACTTGTAA |
| *FOXI1* | GACAAGCGCCTCACTCTCA | CCGGCCTTGCTCTTGTTGTA |
| *GAPDH* | ACACCATGGGGAAGGTGAAG | GTGACCAGGCGCCCAATA |
| *GATA3* | CACGGTGCAGAGGTACCC | AGGGTAGGGATCCATGAAGCA |
| *HES1* | CAACACGACACCGGATAAAC | TGCTCTTCGTCTTTTCTCCA |
| *HES5* | AGCTGCTCAGCCCCAAAGA | TGCTCGATGCTGCTGTTGATG |
| *HES7* | ATCAACCGCAGCCTGGAA | TTCTCCAGCTTCGGGTTCC |
| *HMX3* | TGGTACCCCTACACCCTGAC | CTCTCAGCAAGGCCTTCTCC |
| *KLF4* | CTGCGGCAAAACCTACACAA | CGTCCCAGTCACAGTGGTAA |
| *LFNG* | ACTCCCACCTGGAGAACC | GCGTTCCGCTTGTTTTCAA |
| *MYO7A* | TGAGACCCAGTTTGGCATCA | GGTGTCTCGGTTCTTCTCCA |
| *NANOG* | TGCAGAGAAGAGTGTCGCAAA | GCTGGGTGGAAGAGAACACA |
| *OTX1* | GACCTCCTGCACCCATCC | CAGCTGTGAACGCGTGAA |
| *OTX2* | AGGAGGTGGCACTGAAAATCA | CTGTTGTTGGCGGCACTTA |
| *PAX2* | CGGCTGTGTCAGCAAAATCC | GCTTGGAGCCACCGATCA |
| *PAX8* | GCCCAGTGTCAGCTCCATTA | GCTGTCCATAGGGAGGTTGAA |
| *PHOX2B* | GCCCTGAAGATCGACCTCAC | CGCTCCTGCTTGCGAAA |
| *POU4F3* | CCCCGTACTGCAAGAACC | CATCAAAGCTTCCAAATATATTACCC |
| *POU5F1* | TGGGATATACACAGGCCGATG | GATGGTCGTTTGGCTGAATACC |
| *SALL4* | CACTGGAGAGAAGCCTTTTGTG | CCCCGTGTGTCATGTAGTGA |
| *SOX2* | AGCTCGCAGACCTACATGAA | GGAGTGGGAGGAAGAGGTAAC |
| *SOX3* | GTGTGAAACGGCCCATGAAC | GTGCATCTTGGGGTTCTCCA |
| *SPRY1* | ACCATCCTGTTTGGCCTGTA | GACTAAGCACATGCAGGTTCC |
